# Probing the Crucial Role of Leu31 and Thr33 of the *Bacillus pumilus* CBS Alkaline Protease in Substrate Recognition and Enzymatic Depilation of Animal Hide

**DOI:** 10.1371/journal.pone.0108367

**Published:** 2014-09-29

**Authors:** Nadia Zaraî Jaouadi, Bassem Jaouadi, Hajer Ben Hlima, Hatem Rekik, Mouna Belhoul, Maher Hmidi, Houda Slimene Ben Aicha, Chiraz Gorgi Hila, Abdessatar Toumi, Nushin Aghajari, Samir Bejar

**Affiliations:** 1 Laboratory of Microorganisms and Biomolecules, Centre of Biotechnology of Sfax (CBS), University of Sfax, Sfax, Tunisia; 2 National Leather and Shoe Center (CNCC), Mégrine, Ben Arous, Tunisia; 3 Laboratory for Biocrystallography and Structural Biology of Therapeutic Targets, Molecular and Structural Bases of Infectious Systems, UMR 5086-CNRS-University of Lyon 1, Institute for the Biology and Chemistry of Proteins (IBCP), FR3302, Lyon, France; Oak Ridge National Laboratory, United States of America

## Abstract

The *sapB* gene, encoding *Bacillus pumilus* CBS protease, and seven mutated genes (*sapB*-L31I, *sapB*-T33S, *sapB*-N99Y, *sapB*-L31I/T33S, *sapB*-L31I/N99Y, *sapB*-T33S/N99Y, and *sapB*-L31I/T33S/N99Y) were overexpressed in protease-deficient *Bacillus subtilis* DB430 and purified to homogeneity. SAPB-N99Y and rSAPB displayed the highest levels of keratinolytic activity, hydrolysis efficiency, and enzymatic depilation. Interestingly, and at the semi-industrial scale, rSAPB efficiently removed the hair of goat hides within a short time interval of 8 h, thus offering a promising opportunity for the attainment of a lime and sulphide-free depilation process. The efficacy of the process was supported by submitting depilated pelts and dyed crusts to scanning electron microscopic analysis, and the results showed well opened fibre bundles and no apparent damage to the collagen layer. The findings also revealed better physico-chemical properties and less effluent loads, which further confirmed the potential candidacy of the rSAPB enzyme for application in the leather industry to attain an ecofriendly process of animal hide depilation. More interestingly, the findings on the substrate specificity and kinetic properties of the enzyme using the synthetic peptide para-nitroanilide revealed strong preferences for an aliphatic amino-acid (valine) at position P1 for keratinases and an aromatic amino-acid (phenylalanine) at positions P1/P4 for subtilisins. Molecular modeling suggested the potential involvement of a Leu31 residue in a network of hydrophobic interactions, which could have shaped the S4 substrate binding site. The latter could be enlarged by mutating L31I, fitting more easily in position P4 than a phenylalanine residue. The molecular modeling of SAPB-T33S showed a potential S2 subside widening by a T33S mutation, thus suggesting its importance in substrate specificity.

## Introduction

Leather processing is one of the oldest industries known to mankind. Despite its significant contributions to the socio-economic development of several countries around the world, this industry has been the target of mounting criticism owing to the pollution it causes to the environment. It has, therefore, been under pressure to comply with increasingly stringent global environmental regulations. In fact, the conventional leather making process includes a complex set of operations, such as pre-tanning, tanning, post tanning, and finishing, which involve the application of various hazardous chemicals, notably lime and sodium sulphide, that generate several environmental and waste disposal problems. The application of those chemicals can also lead to the destruction of the hair, thus causing high biological oxygen demand (BOD), chemical oxygen demand (COD), and total suspended solid (TSS) loads in the effluents. The search for cleaner technologies that can help overcome the serious problems associated with the conventional depilating methods has, therefore, become a necessity in the leather industry [1].

In order to overcome the hazards caused by these effluents, enzymes have often been proposed as viable alternatives. In fact, enzymes have long been used as alternatives to chemicals to improve the efficiency, safety, and cost-effectiveness of a wide range of industrial systems and processes [2]. Currently, the most commonly used biotechnological applications cover all the stages of leather making and waste treatment processes. An emerging technology is the use of proteolytic enzymes in the depilation process, which minimizes/replaces the use the major pollutant in the tannery process, namely sulphide.

Microbial alkaline proteases are among the most important hydrolytic enzymes. They play important roles in both cellular metabolic processes and industrial sectors, accounting for more than 65% of the global industrial enzyme sales [3]. Alkaline proteases produced from *Bacillus* can withstand high temperature, pH, chemical denaturing agents, and non-aqueous environments. They have attracted considerable attention particularly due to their promising potential for application in a wide range of industries, including the detergent, leather, and pharmaceutical industries. The keratinolytic activity of these enzymes has also been of interest in several biotechnological processes, such as the management of waste from various food-processing industries, the recovery of silver from used X-ray and photographic films, and production of proteinaceous fodder from waste feathers or keratin-containing materials [4].

Due to their efficiency, low cost, and eco-friendliness, microbial alkaline proteases have attracted increasing attention for application in enzymatic depilation as substitutes to chemical agents, such as sodium sulphide, lime, and chromate, which have long been used in conventional industrial depilation processes. Several researchers have, therefore, focused on the isolation and characterization of novel microbial strains with efficient depilation activity. Alkaline proteases/keratinases selectively cleave the hair on the skin without causing damage to the skin, thus leaving the quality of the leather unaffected [5], [6]. The adequacy of keratinolytic proteases with mild elastinolytic but no collagenolytic activities for the depilation process has also been highlighted in the literature. Keratinases may participate in the selective breakdown of the keratin tissues in hair follicles, thus pulling out intact hairs without affecting the ductile strength of the leather [4].

Keratinases are robust enzymes that are active over a wide range of temperature and pH. The sequence analysis of keratinases indicates their close relatedness to subtilisin and serine proteases, which are generally from bacterial origins. They are specific to aliphatic or hydrophobic amino-acid residues, most active at around pH 10, and with a molecular weight ranging from 10 to 35 kDa. This class of proteases also has a tight relationship with subtilisins isolated from *Bacillus* strains, exhibiting similar protein sequences and biochemical characteristics. Various keratinases have been produced from *Bacillus* species, including *B. licheniformis*, *B. pumilus*, *B. subtilis*, *B. circulans*, and *Brevibacillus brevis* [4], [7], [8].

Although several subtilisins, including keratinases, have been described in the literature, little data is currently available on the distinction between the two types of hydrolytic enzymes. In fact, while several subtilisins display efficient keratinase activity, some others either have a very low or no keratinase activity. Nevertheless, little work has so far been performed on the modes or mechanisms of actions and structural differences between those two types of proteases. Moreover, so far and to the authors’ knowledge, no previous work has been performed to investigate the conversion of a subtilisin into a keratinase or *vice versa*. In this context, the authors have previously reported the purification and characterization of a serine protease, called SAPB, from *B. pumilus* CBS. The pure enzyme showed optimal activity at pH 10.6 and 65°C. The *sapB* gene was cloned and expressed in *Escherichia coli* [9]. The laundry detergent stability and dehairing ability of SAPB were also investigated [10]. Furthermore, the effects of five essential amino-acid residues on the biochemical properties of the enzyme were also explored, allowing the generation of seven efficient and thermostable mutant enzymes, particularly SAPB-N99Y, SAPB-L31I/N99Y, SAPB-T33S/N99Y, and SAPB-L31I/T33S/N99Y. Among those mutants, and compared to the wild-type enzyme, the triple mutant was noted to display the highest levels of specific activity, catalytic efficiency and stability at elevated pH and temperature values with casein as a substrate [11]. The authors have also reported on the overexpression of *sapB* and *sapB*-L31I/T33S/N99Y genes in *B. subtilis* DB430 [12]. The present study aimed to investigate the overexpression of all SAPB mutant enzymes in *B. subtilis* DB430. It also explored their substrate specificities and potential keratin biodegradation and depilation activities. The effects of L31I and T33S mutations on their animal hide depilation ability and the relationship between keratin/casein activity ratios were also examined.

## Materials and Methods

### 2.1. Substrates and Chemicals

Unless otherwise specified, all substrates, chemicals and reagents were of analytical grade or highest available purity, and purchased from Sigma Chemical Co. (St. Louis, MO, USA).

### 2.2. Bacterial Strains, Plasmids, Media, and Cultivation Conditions

The two protease-deficient host strains used were the *B. subtilis* DB430 strain (*trpC npr apr epr bpf ispl*), a generous gift from Dr. Philippe Glaser and Dr. Marie Françoise Hullo (LGMP, Institut Pasteur, Paris), and the *E. coli* DH5α strain (Invitrogen, Carlsbad, CA, USA). The *B. pumilus* CBS strain [10] was used as a donor for the SAPB enzyme and *sapB* wild-type gene. The plasmid pBJ4, containing the *sapB* wild-type gene [9], and plasmids pBJ22, pBJ25, pBJ28, pBJ31, pBJ34, pBJ37 and pBJ40, containing *sapB*-L31I, *sapB*-T33S, *sapB*-N99Y, *sapB-*L31I/T33S, *sapB*-L31I/N99Y, *sapB-*T33S/N99Y, and *sapB-*L31I/T33S/N99Y, respectively, were used as sources for mutated *sapB* genes [11]. The *E. coli*-*Bacillus* shuttle vector pBSMuL2, kindly provided by Dr. Thorsten Eggert (Institut fur Molekulare Enzymtechnologie, Heinrich-Heine Universität Dusseldorf, Julich, Germany), was used to construct the expression plasmid in *E. coli* DH5α. The pNZ1 and pNZ2 plasmids, used for the production of rSAPB and SAPB-L31I/T33S/N99Y proteins, respectively, were previously described elsewhere [12]. The different strains harboring the wild and mutant type genes were routinely cultured in LB media consisting of (g.l^−1^): peptone, 10; yeast extract, 5; and NaCl, 5 at pH 7.4. Skimmed milk media were used for the screening of protease-producing recombinant strains. The production medium consisted of a 2 × LB and 10 g.l^−1^ glucose at pH 7.4 [12]. The media were autoclaved for 20 min at 120°C and supplemented, when required, with antibiotics at the following concentrations: Ampicillin (100 µg.ml^−1^) for *E. coli*; and Kanamycin (5 µg.ml^−1^) for *B. subtilis*.

### 2.3. DNA Manipulation and Sequencing

Plasmid DNA was isolated from *E. coli* and *B. subtilis* using general molecular biology techniques as described by Sambrook et al. [13]. PCRs were performed using an Applied Biosystems 2720 thermal cycler. The amplification reaction mixtures (50 µl) contained 20 pg of each primer, 200 ng of DNA template, amplification buffer, and 2 U of *Pyrococcus furiosus* DNA polymerase (Biotools, Madrid, Spain). The cycling parameters were as follows: 94°C for 3 min, followed by 35 cycles of 94°C for 30 s denaturation, 54°C for 60 s primer annealing, and 72°C for 120 s extension). The PCR products were then purified using an agarose gel extraction kit (Jena Bioscience, GmbH, Germany). DNA sequencing was carried out by an automated DNA sequencer ABI Prism 3100**-**Avant Genetic Analyser (Applied Biosystems, Foster City, CA, USA) with the Big-Dye terminator cycle sequencing kit recommended by the manufacturer (Amersham Pharmacia Biotech, Buckinghamshire, UK).

### 2.4. Construction of Protease Overexpression Plasmids and *Bacillus* Transformation

To overproduce SAPB mutated proteases in *B. subtilis* DB430, a 1260 bp *Eco*RI/*Hind*III DNA fragment from pBJ22, pBJ25, pBJ28, pBJ31, pBJ34, and pBJ37 plasmids carrying the whole *sapB*-L31I, *sapB*-T33S, *sapB*-N99Y, *sapB*-L31I/T33S, *sapB*-L31I/N99Y, and *sapB*-T33S/N99Y genes, respectively, were subcloned in the pBSMuL2 (7559 bp) shuttle vector linearized by *Eco*RI/*Hind*III to produce pNZ7 (8819 bp), pNZ8 (8819 bp), pNZ9 (8819 bp), pNZ10 (8819 bp), pNZ11 (8819 bp), and pNZ12 (8819 bp) plasmids, respectively.

*B. subtilis* DB430 was transformed as previously described by Zaraî Jaouadi et al. [12]. After the suitable dilution of competent cells, the pNZ7, pNZ8, pNZ9, pNZ10, pNZ11, and pNZ12 plasmids or pBSMuL2 plasmid DNA multimers were added, and the samples were incubated at 37°C for 20 min. Transformation mixtures were then spread on LB-Kanamycin agar. Kanamycin resistant colonies the *B. subtilis* DB430 transformants were screened for protease activity on LB-Kanamycin-skimmed milk plates, based on the detection of cleared zone formations around *B. subtilis* DB430 transformant colonies. The latter were further screened by PCR to confirm the introduction of the *sapB* genes.

### 2.5. Enzyme Activity Assays

The keratinolytic activity of the recombinant SAPB enzymes was determined under the optimal pH and temperature values of the respective enzymes using keratin azure as a substrate [7]. One keratin unit was defined as the amount of enzyme causing an increase of 0.1 in absorbance at 440 nm in one min. The same protocol was used to determine enzyme activity on azo-casein.

Caseinolytic activity was measured using the Folin-Ciocalteu method [11], with Hammersten casein (Merck, Darmstadt, Germany) as a substrate. One casein unit was defined as the amount of enzyme that produced 1 µg of amino-acid equivalent to tyrosine per min. The same protocol was used to determine enzyme activity on natural proteins, namely keratin, elastin-orcein, and albumin.

### 2.6. Enzyme Purification Procedure

The SAPB enzymes were purified following the procedures previously described by Zaraî Jaouadi et al. [12]. In brief, five hundred ml of a 72-h-old culture of *B. subtilis* DB430 harboring the pNZ1, pNZ2, pNZ7, pNZ8, pNZ9, pNZ10, pNZ11, or pNZ12 plasmids were harvested by centrifugation at 10,000×*g* for 35 min to remove microbial cells. For each culture, the cell-free supernatant containing extracellular protease was used as the crude enzyme preparation and submitted to the following purification steps. Each supernatant was heat-treated at 65°C for 15 min, and insoluble material was removed by centrifugation at 10,000×*g* for 30 min. The clear supernatant was precipitated between 40% and 60% ammonium sulphate saturation. The precipitate was then recovered by centrifugation at 14,000×*g* for 20 min, resuspended in a minimal volume of 50 mM 2-(*N*-morpholino) ethanesulfonic acid (MES) buffer containing 2 mM CaCl_2_ at pH 5.5 (Buffer A), and dialyzed overnight against repeated changes of buffer A. Insoluble material was removed by centrifugation at 14,000×*g* for 20 min. The supernatant was loaded on a fast performance liquid chromatography (FPLC) system using an UNO S-1 column (Bio-Rad Laboratories, Inc., Hercules, CA, USA) equilibrated in buffer A. The column (7 mm×35 mm) was rinsed with 500 ml of the same buffer. After being washed with the same buffer A, the unadsorbed protein fractions were eluted. Adsorbed material was eluted with a linear NaCl gradient (from 0 to 0.5 M in buffer A) at a rate of 60 ml.h^−1^. Fractions with protease activity were pooled and applied to high performance liquid chromatography (HPLC) system using a Bio-Sil SEC 125–5 column (7.8 mm×300 mm), pre**-**equilibrated with 25 mM HEPES buffer and supplemented with 2 mM CaCl_2_ and 150 mM NaCl at pH 7.5 (Buffer B). Proteins were separated by isocratic elution at a flow rate of 30 ml.h^−1^ with buffer B and detected using a UV**-**VIS detector (Knauer, Berlin, Germany) at 280 nm. The pooled fractions (eluted at a void volume of 1.8), exhibiting protease activity and a retention time of 20 min, were concentrated in centrifugal micro**-**concentrators (Amicon Inc., Beverly, MA, USA) with 10 kDa cut-off membrane and stored at –20°C for further analysis.

### 2.7. Protein Measurement and Analytical Methods

Protein concentration was determined by the method of Bradford [14], using a Dc protein assay kit purchased from Bio-Rad Laboratories (Inc., Hercules, CA, USA) with bovine serum albumin as a reference. Analytical soduim dodecyl sulphate polyacrylamide gel electrophoresis (SDS-PAGE) was performed following the method of Laemmli [15]. Protein bands were visualized with Coomassie Brilliant Blue R-250 staining. Casein-zymography analysis was performed as previously described elsewhere [9]. Low molecular weight markers from Amersham Biosciences were used as protein marker standards. The molecular masses of the selected purified SAPB-L31I, SAPB-T33S, and SAPB-N99Y enzymes were analyzed in the linear mode by MALDI-TOF/MS using a Voyager DE-RP instrument (Applied Biosystems/PerSeptive Biosystems, Inc., Framingham, MA, USA). Bands of purified enzymes (SAPB-L31I, SAPB-T33S, and SAPB-N99Y) were separated on SDS gels and transferred to a ProBlott membrane (Applied Biosystems, Foster City, CA, USA). N-terminal sequence analyses were performed by automated Edman’s degradation using an Applied Biosystem Model 473A gas-phase sequencer.

### 2.8. Substrate Specificities and Kinetic Studies

The substrate specificity of rSAPB (pH 10.6, 65°C), SAPB-L31I (pH 11.5, 65°C), SAPB-T33S (pH 12, 65°C), SAPB-N99Y (pH 11, 75°C), SAPB-L31I/T33S (pH 12, 65°C), SAPB-L31I/N99Y (pH 11.5, 70°C), SAPB-T33S/N99Y (pH 12, 70°C), and SAPB-L31I/T33S/N99Y (pH 12, 70°C) were determined using natural (keratin, casein, elastin-orcein, and albumin) and modified (keratin azure, azo-casein, and collagen types I and II FITC conjugate) proteins as well as synthetic para-nitroanilide (pNA) linked peptide chromogenic substrates. The synthetic substrates were Succinyl-XXX or XXXX-pNA with XXX or XXXX representing YLV, AAV, AAI, AAL, AAA, APA, and AAF or FAAF, AAPF, LLVY, AAPM, AAPL, and AAVA. Peptides were dissolved in 10% (v/v) dimethylformamide (DMF) before being diluted in buffer, and prepared just before the experiment since nitroanilide substrates show varying degrees of autolysis during storage.

Kinetic parameters were calculated from the initial rate activities of the eight purified SAPB enzymes using YLY and FAAF as synthetic peptide substrates at different concentrations, ranging from 0.2 mM to 50 mM. Michaelis–Menten constant (*K*_m_) and maximal reaction velocity (*V*_max_) values were calculated by Lineweaver-Burk plots using Hyper32 software.

The pH and temperature values used in this study were adjusted to the optimum conditions for each SAPB enzyme as reported elsewhere [11]. Enzymatic activities were determined on each substrate according to standard conditions. For the natural substrate, the complete hydrolysis of a protein unit was defined as the amount of enzyme that produced 1 µg of amino-acid equivalent to tyrosine per min, in absorbance at 660 nm, under the experimental conditions described. For the modified substrate, one protein azure unit was defined as the amount of enzyme causing an increase of 0.1 in absorbance at 440 nm in one min under the assay conditions described. Collagenolytic activities were determined by measuring absorbance at 440 nm as illustrated in the protocol of Sigma-Aldrich Co. LLC. Activity with pNA substrates was tested at suitable pH and temperature for 10 min in 100 mM buffer containing 2 mM Ca^2+^. For the synthetic peptide substrate, the amount of released pNA was recorded at 405 nm. The initial rates measured as A405/min were converted into velocity (µM^−1^.min^−1^) for each substrate concentration using the molar absorbance coefficient for pNA (9800 M^−1^.cm^−1^ at 405 nm). One unit of enzymatic activity was defined as the amount of enzyme releasing 1 µmole of pNA under standard assay conditions.

### 2.9. Zymorgam Gel Analysis

To confirm the substrate specificity profile of SAPB enzymes, discontinuous substrate native-PAGE (Zymogram analysis) was performed with a 4% stacking gel, except that 1 mg/ml keratin or casein, as a substrate, were incorporated into the 10% separation gel. Electrophoresis was performed at a constant current of 25 mA under non-reducing conditions. The gels were then gently washed and incubated at 50°C for 2 h in 100 mM Glycine**-**NaOH buffer at pH 10 supplemented with 2 mM CaCl_2_, which produced a keratin or casein cleared zone at the location of the proteolytic band of each SAPB enzyme. A clear zone was visualized by fixing the gel with ice**-**cold trichloroacetic acid 20% (w/v) for 1 h, staining it with 0.1% Coomassie Brilliant Blue G**-**250 (Bio-Rad Laboratories, Inc., Hercules, CA, USA) in water/methanol/acetic acid 60∶30∶10, and distaining it in the same solution without dye.

### 2.10. Determination of Hydrolysis Degree

Keratin hydrolysis was carried out according to Zaraî Jaouadi et al. [7] at 65°C and pH 10.6 (for rSAPB); 65°C and pH 11.5 (for SAPB-L31I); 65°C and pH 12 (for SAPB-T33S), 75°C and pH 11 (for SAPB-N99Y), 65°C and pH 12 (for SAPB-L31I/T33S), 70°C and pH 11.5 (for SAPB-L31I/N99Y), 70°C and pH 12 (for SAPB-T33S/N99Y), and 70°C and pH 12 (for SAPB-L31I/T33S/N99Y). An amount of 4 g of keratin azure was dissolved in 100 ml of assay buffer and then treated with 2,000 U of the purified target SAPB enzymes.

### 2.11. Enzyme Preparation for Leather Processing

The cell-free supernatant of a 72-h-old culture of *B. subtilis* DB430 harboring the pNZ1 plasmid was brought to 80% (w/v) saturation with ammonium sulphate. The precipitated proteins were centrifuged at 14,000 × g for 20 min at 4°C. The resultant pellet was dissolved in buffer B and dialyzed against the same buffer. The dialyzed semi-purified enzyme was used for depilation in leather processing.

### 2.12. Hide-Depilation Ability of SAPB Enzymes

Small pieces (about 6 cm × 6 cm) of haired goat, rabbit, bovine, and sheep skins, which were freshly obtained from a local municipal slaughterhouse (Sfax municipal slaughterhouse, permission was obtained from this slaughterhouse to use these animal parts), and rinsed to remove excess blood, were placed into 20 ml of buffer B containing a purified rSAPB, SAPB-L31I, SAPB-T33S, SAPB-N99Y, SAPB-L31I/T33S, SAPB-L31I/N99Y, SAPB-T33S/N99Y, or SAPB-L31I/T33S/N99Y enzymes having 2,000 U of keratinase activity. After 8 h of incubation at 37°C, the skins were taken out and the hair was gently hand-pulled to test whether it had parted from the skin. Since, to the authors’ knowledge, no quantitative method is currently available for the determination of depilation effects, this ability was defined qualitatively as “no”, “yes” or “easily”. Depilation efficiency was assessed according to the depilated area of the skin at the end of the process, and the quality of the depilated skin was estimated according to naked-eye observations and microscopic examinations made after treatments.

The handling of the skin from rabbit, goat, bovine and sheep animals were carried out in strict accordance with the recommendations in the Guide for the Care and Use of Laboratory Animals issued by the University of Sfax, Tunisia. The protocol was approved by the Tunisian Committee on the Ethics of Animal Experiments.

### 2.13. Semi-Industrial Scale Test for Goat Skin Depilation and Scanning Electron Microscopy Analysis

The depilation activity of rSAPB enzyme was tested in the “SO. SA. CUIR” leather tannery (M’Saken, Sousse, Tunisia) using a 100 kg fresh goat hides obtained from a local slaughterhouse. Wet salted goat hides were used for depilating experiments. The experiments were performed in a mini drum (cylindrical rotating reactor, used for hide and leather processing) with shaking at 15 rpm. The skins were cut into two halves along the backbone. The right half was used for enzymatic depilation; the left half was used for conventional lime and sulphide-based depilation and served as a control. Prior to depilation, the skins were washed and soaked in water (200% v/wt) for 5 h with intermittent changing to remove salt, dirt, and blood. The soaked weight of all the left and right halves were recorded, and the percentages of chemicals and enzyme used in the experiment was based on this soaked weight. For the conventional process, the paste method was used wherein 10% lime and 2% sodium sulphide were mixed with 10% water, and the thus prepared paste was applied on the flesh side (Sivasubramanian et al., 2008a; Sivasubramanian et al., 2008c). It was left overnight at room temperature and then depilated using the conventional method. For the enzymatic group, the optimized dip method was adopted wherein the soaked skins were dipped in water float (100% v/wt) containing 2,000 U enzyme. The float was left for 6 h and then depilated by conventional methods. After depilation, both the chemical and depilated enzyme-treated pelts were processed and finished as dyed crusts as per conventional procedures. The depilated skins were visually assessed, and the quality of the removed hair was studied by a light microscope.

Samples were cut from depilated pelts, and then washed and fixed in formal saline. They were then dehydrated using a graded ethanol series and freeze-dried. The dried samples from goat leather treated with rSAPB were cut into approximately 5 mm thick slices and fixed onto metallic sample holders with conducting silver glue and then sputtered with a layer of gold (Edwards E-306). The micrographs were then observed using a scanning electron microscope (SEM) (XL 30 ESEM with an integrated EDAX system, Philips, Netherlands) operating at an accelerating voltage of 12 kV. The dyed crust samples were cut and directly coated with gold for SEM analysis.

### 2.14. Assessment of the Physical and Chemical Properties of Dyed Crust

The dyed crusts from both the rSAPB-treated and chemically-treated groups were visually assessed for quality and tested for strength characteristics as per standard procedures. After conditioning at room temperature and relative humidity of 65% for 48 h, the properties of the crust leather, including tensile strength, elongation at break, and tear strength were measured using standard methods [16]. The samples were also assessed for general appearance and dyeing characteristics. The Cr_2_O_3_ content was determined as per the standard procedure [17]. The crusts were also analyzed for their hide substance and grease content [18], [19].

### 2.15. Measurements of Pollution Load

To assess the effect of enzymatic depilation on pollution load, effluents were collected at the end of the depilation of the rSAPB-treated and control groups and analyzed in terms of well-established pollution parameters, *viz*. BOD, COD, TSS, sulphide, calcium, conductivity, and pH following standard analytical procedures [20].

### 2.16. Bioinformatics and Homology Modeling

The automated comparative protein structure homology modeling server, SWISS-MODEL (http://www.expasy.org/swissmod/), was used to generate the three-dimensional model of SAPB enzymes based on the crystal structure of subtilisin E (PDB-code 1SCJ). The Deep View Swiss-PDB Viewer software from the EXPASY server (http://www.expasy.org/spdbv) and PyMOL v0.99 (http://www.pymol.org) were used to visualize and analyse the three-dimensional model.

### 2.17. Statistical Analysis

All data were analyzed using Microsoft Excel. Values are expressed as means ± standard deviation of results from three independent experiments. Data were considered as statistically significant for *P* values of less than or equal to 0.05.

## Results and Discussion

### 3.1. Overexpression of *sapB* Mutated Genes in Protease-Deficient *Bacillus Subtilis* DB430

The plasmid constructs pNZ7, pNZ8, pNZ9, pNZ10, pNZ11, and pNZ12 in which the *sapB*-L31I, *sapB*-T33S, *sapB*-N99Y, *sapB*-L31I/T33S, *sapB*-L31I/N99Y, and *sapB*-T33S/N99Y genes were inserted between the restriction sites for *Eco*RI/*Hin*dIII at the multiple cloning site of pBSMuL2 were prepared in *E. coli*, respectively. To guarantee the efficient DNA uptake for the naturally competent *B. subtilis* DB430 cells, multimeric plasmid DNA forms of generated plasmids and pBSMuL2 were constructed by the *in vitro* ligation of the linearized plasmids and used for transformation. Unlike the Kanamycin resistant colonies of *B. subtilis* transformed with pBSMuL2, those transformed with plasmid constructs showed clear zones of casein hydrolysis. This was correlated with the PCR detection of the *sapB* genes being introduced. The recombinant plasmids were identified through restriction enzyme analysis and DNA sequencing [12].

### 3.2. Production of Recombinant and Mutated SAPB Enzymes

The production of SAPB enzymes was noted to start after a 6-h lag phase and then to increase exponentially and concomitantly with the cellular growth increases by *B. subtilis* DB430/pNZ1, pNZ2, pNZ7, pNZ8, pNZ9, pNZ10, pNZ11, and pNZ12. The SAPB-N99Y enzyme from *B. subtilis* DB430/pNZ9 was noted to exhibit the highest keratinolytic activity of 18,000 U.ml^−1^, with 4,500 U.ml^−1^ of caseinolytic activity, followed by rSAPB (13,750 U.ml^−1^ of keratinolytic activity and 5,500 U.ml^−1^ of caseinolytic activity). Interestingly, the levels of protease production that were obtained by the recombinant strains of *B. subtilis* DB430/pNZ9 and *B. subtilis* DB430/pNZ1 were 21 and 37-fold higher than those obtained by strains of *E. coli* DH5α/pBJ28 (210 U.ml^−1^) and *E. coli* DH5α/pBJ4 (150 U.ml^−1^), respectively, using casein as a substrate [11]. Unlike the case of the wild-type strain [9], this production proved to be highly reproducible, and its efficiency could presumably be attributed to the strong P_59_ constitutive promoter carried by the pBSMuL2 shuttle vector.

### 3.3. Purification and Identification of SAPB Enzymes

The results of the specific activities of the purified recombinant and mutated SAPB enzymes at the last purification step are summarized in Table 1. The findings revealed that the rSAPB and SAPB mutated proteases overexpressed in *B. subtilis* exhibited specific activities ranging from 24,950 U.mg^−1^ (for rSAPB) to 45,500 U.mg^−1^ (for SAPB-L31I/T33S/N99Y) when casein was used as a substrate. In fact, these results are similar to those reported for the rSAPB and SAPB mutated enzymes expressed in *E. coli* whose specific activities were 25,500 U.mg^−1^ and 46,310 U.mg^−1^, respectively [11]. Moreover, the SAPB mutated proteases overexpressed in *B. subtilis* were noted to exhibit specific activities ranging from 15,469 U.mg^−1^ (for SAPB-L31I) to 139,012 U.mg^−1^ (for SAPB-N99Y) when keratin was used as a substrate.

**Table 1:** Specific activities and keratin/casein ratios of the purified wild-type and mutant SAPB enzymes using keratin and casein as substrates.

SAPB enzyme	*Bacillus subtilis* DB430	Specific activity with keratin (U.mg^−1^)^a^^,^ b	Relative specific activity to wild-type with keratin^c^	Specific activity with casein (U.mg^−1^)^a^^,^ b	Relative specific activity to wild-type with casein^c^	Keratin/casein ratio
WT	pNZ1	62375±820	1.00	24950±640	1.00	2.50
L31I	pNZ7	15469±357	0.25	28125±663	1.12	0.55
T33S	pNZ8	19420±415	0.28	31321±668	1.25	0.62
N99Y	pNZ9	139012±999	2.22	34753±656	1.39	4.00
L31I/T33S	pNZ10	26345±643	0.34	37634±670	1.50	0.70
L31I/N99Y	pNZ11	54689±781	0.87	38911±684	1.55	1.40
T33S/N99Y	pNZ12	52340±770	0.83	40261±707	1.61	1.30
L31I/T33S/N99Y	pNZ2	29052±665	0.46	45500±755	1.82	0.63

a Specific activity is defined as units (U) of activity per amount (mg) of protein. 1 U of protease activity was defined as the amount of enzyme that liberated 1 µg tyrosine per min under the optimal temperature and pH values of the respective recombinant enzymes using keratin or casein as a substrate. Proteins were estimated by the Bradford method using the Dc protein assay kit obtained from Bio-Rad Laboratories (Inc., Hercules, CA, USA).

b The experiments were conducted three times and ± standard errors are reported.

c The relative activity is calculated by taking the specific activity of the wild-type as 1.00.

The SDS-PAGE analysis of the pooled fractions of each purified SAPB enzyme overexpressed in the *B. subtilis* system showed a single band corresponding to an apparent molecular mass of about 34 kDa [12]. The exact molecular masses of the SAPB-L31I, SAPB-T33S, and SAPB-N99Y mutant enzymes from the *B. subtilis* DB430/pNZ7, *B. subtilis* DB430/pNZ8, and *B. subtilis* DB430/pNZ9 strains were confirmed by MALDI-TOF mass spectrometry as being 34579.10 Da, 34585.54 Da, and 34601.41 Da, respectively, which are similar to those previously reported for the same proteases expressed in the *E. coli* system [11]. Zymographic analysis also revealed one zone of caseinolytic activity for the purified sample co-migrating with proteins whose molecular masses were of approximately 34 kDa [12]. Taken together, these findings indicate that the heterologous SAPB enzymes overexpressed in *B. subtilis* DB430 are monomeric proteins comparable to those previously reported for SAPB proteases expressed in *E. coli* [11] and for SAPB produced by *B. pumilus* CBS [9].

The N-terminal sequencing of the blotted purified SAPB-L31I, SAPB-T33S, and SAPB-N99Y enzymes from the *B. subtilis* DB430/pNZ7, *B. subtilis* DB430/pNZ8, and *B. subtilis* DB430/pNZ9 strains allowed the identification of the first 21, 23, and 25 amino-acid residues, namely AQTVPYGIPQIKAPAVHAQGY, AQTVPYGIPQIKAPAVHAQGYKG, and AQTVPYGIPQIKAPAVHAQGYKGAN, respectively. These sequences showed uniformity, indicating that they were isolated in a pure form. The findings also revealed that the N-terminal amino-acid sequences of SAPB-L31I, SAPB-T33S, and SAPB-N99Y from the *B. subtilis* system were completely identical to those of the same SAPB enzymes expressed in the *E. coli* system [11].

### 3.4. Substrate Specificity Profiles of SAPB Enzymes

The substrate specificity of proteases is often attributed to the amino-acid residues preceding the peptide bond they hydrolyze. The relative hydrolysis rates of various protein substrates were investigated to elucidate the amino-acid preference/substrate specificity of SAPB enzymes (Table 2). Among the proteinaceous substrates tested, rSAPB and SAPB-N99Y were noted to show highest activity with keratin and keratin azure, followed by elastin. However, a poor to moderate hydrolysis of casein and albumin was observed. For the other recombinant SAPB enzymes, on the other hand, the highest activities were observed with casein and azo-casein. While a relatively high rate of hydrolysis was observed with keratin and keratin azure, no collagenase activities were detected on collagen types I and II, which provided further support for the relevance of SAPB enzymes for hair removal in the leather industry. In fact, the lack of collagenase activity is highly valued in the leather industry because this advantage would reduce the potential degradation of collagen, the major leather-forming protein. This criterion was particularly satisfied by rSAPB and SAPB-N99Y, which highlights their suitability for animal hide depilation.

**Table 2:** Substrate specificities of the wild-type and mutant SAPB enzymes with proteins and synthetic peptides as substrates.

Substrate	Relative activity (%)^a^^,^ b							
	WT	L31I	T33S	N99Y	L31I/T33S	L31I/N99Y	T33S/N99Y	L31I/T33S/N99Y
*Natural protein (40 g.l^−1^ at 660 nm)*								
Keratin	100±2.5	58±1.5	61±1.5	100±2.5	55±1.5	100±2.5	100±2.5	75±2.0
Elastin-orcein	52±1.4	45±1.3	52±1.4	60±1.6	42±1.2	48±1.3	50±1.4	40±1.2
Albumin	30±1.2	33±1.2	40±1.2	38±1.2	30±1.2	52±1.4	60±1.5	50±1.4
Casein	45±1.3	100±2.1	100±2.1	19±1.0	100±2.1	85±2.1	76±2.0	100±2.5
*Modified protein (40 g.l^−1^at 440 nm)*								
Keratin azure	100±2.5	67±1.6	72±1.9	100±2.5	60±1.5	100±2.5	100±2.5	91±2.3
Azo-casein	37±1.2	100±2.5	100±2.5	15±0.9	100±2.5	73±1.9	81±2.1	100±2.5
Collagen type I	0±0.0	0±0.0	0±0.0	0±0.0	0±0.0	0±0.0	0±0.0	0±0.0
Collagen type II	0±0.0	0±0.0	0±0.0	0±0.0	0±0.0	0±0.0	0±0.0	0±0.0
*Synthetic peptide (10 mM at 405 nm)*								
YLV	100±2.5	10±0.7	24±1.1	100±2.5	19±1.0	72±2.0	70±1.9	17±1.0
AAV	98±2.5	40±1.3	44±1.3	97±2.5	42±1.3	80±2.1	81±2.1	41±1.3
AAI	92±2.3	37±1.2	41±1.3	95±2.4	35±1.2	100±2.5	100±2.5	38±1.2
AAL	86±2.2	32±1.2	37±1.2	90±2.3	32±1.2	90±2.3	85±2.1	31±1.2
AAA	77±2.0	28±1.2	33±1.2	81±2.1	28±1.2	70±1.9	67±1.8	26±1.1
APA	60±1.1	25±1.1	30±1.2	64±1.7	26±1.1	57±1.5	51±1.4	24±1.1
AAF	51±1.4	30±1.2	35±1.2	60±1.1	30±1.2	55±1.5	47±1.4	29±1.2
FAAF	11±0.7	100±2.5	100±2.5	10±0.7	100±2.5	77±2.0	80±2.1	100±2.5
AAPF	12±0.8	99±2.5	97±2.5	11±0.7	98±2.5	49±1.4	38±1.2	96±2.5
LLVY	15±0.9	97±2.5	95±2.4	12±0.9	96±2.5	31±1.2	30±1.2	93±2.4
AAPM	24±1.1	80±2.1	81±2.1	20±1.0	78±2.0	17±1.0	15±0.9	82±2.1
AAPL	35±1.2	60±1.5	66±1.8	33±1.2	57±1.5	25±1.1	23±1.1	58±1.5
AAVA	44±1.3	52±1.4	59±1.5	42±1.3	48±1.3	38±1.2	37±1.2	50±1.4

a Values represent means of three replicates, and ± standard errors are reported.

b The unit activity of each substrate was determined by measuring absorbance at specified wavelengths as described in Section 2.

The preferences of the SAPB enzymes for synthetic substrates incorporating N-terminal residues to the cleavage site (P1, P2, etc.) have also been elucidated. The Suc-P4-P3-P2-P1-pNA substrate where, according to the nomenclature of Schechter and Berger [21], Suc is a succinyl group and Pn represents the individual amino-acids, was abbreviated to the 4 core amino-acid residues. The amino-acids at position P1 exerted strong effects on the catalytic action of the SAPB enzymes, see Table 2. The order of the substrate specificity values of rSAPB and SAPB-N99Y enzymes was almost the same *i.e.*, YLV ≈ AAV ≈ AAI ≈ AAL>AAA>APA>AAF>AAPL>AAVA>AAPM>LLVY>AAPF and FAAF. Hence, the rSAPB and SAPB-N99Y enzymes showed preference for hydrophobic aliphatic amino-acids (valine, isoleucine, and leucine) at position P1 and exhibited the highest activity for YLV, AAV, AAI, and AAL. By analogy, most of the amino-acids at position P1 in the native keratin could be assumed to be aliphatic residues in nature. This preference for aliphatic amino-acids could presumably be due to the active site cleft lined up with aliphatic amino-acid residues.

Furthermore, low or very low hydrolysis was detected when Met, Ala or Tyr where present at the P1 position. A decrease in the hydrolysis rate was, however, observed for FAAF, and AAPF, indicating that catalytic activity was also affected by the amino-acid in position P2. When Suc-(Ala)n-pNA was used, a minimum length of two residues was required for hydrolysis. Enzymatic activity was largely dependent on secondary enzyme substrate contacts with amino-acid residues (P2, P3, etc.) more distant from the scissile bond, as illustrated by the differences observed between the activity of YLV and AAVA, a quality that was previously demonstrated for microbial keratinases KERUS [7], KERAB [22], and SAPDZ [8]. The SAPB-L31I, SAPB-T33S, SAPB-L31I/T33S, and SAPB-L31I/T33S/N99Y enzymes, on the other hand, showed preference for aromatic amino-acid residues, such as Phe and Tyr, and the carboxyl side of the splitting point in the P1 and P4 positions of pNA substrates. They were, therefore, active against phenylalanine (FAAF and AAPF) and tyrosine (LLVY) peptide bonds. Their substrate specificity profile suggested that they largely preferred hydrophobic substrates, especially those with aromatic residues occupying the P1 and P4 positions, which were almost the same *i.e.*, FAAF ≈ AAPF ≈ LLVY>AAPM>AAPL>AAVA>AAV>AAI>AAL>AAF>AAA>APA and YLV. The nature of the amino-acid in position P2 also influenced the specificity in position P1. This could be noted from the differences in the activity of the FAAF and APA substrates; a decrease in activity was observed when proline was substituted by alanine, thus confirming the effect of the amino-acid present at position P2. These characteristics, also reported for other subtilisins from *Bacillus* origins [23], [24], indicated that the SAPB-L31I, SAPB-T33S, SAPB-L31I/T33S, and SAPB-L31I/T33S/N99Y proteases were closely similar to subtilisins not only in terms of specificity for position P1 but also with regard to the effects of amino-acids residues neighboring the cleavage site. Nevertheless, some differences were observed in side chain specificity at P2, which could presumably indicate the presence of an extended active site. Proline was also noted to promote hydrolysis at the P2 position in these SAPB proteases, a feature that was not observed for subtilisin E [24].

### 3.5. Kinetic Parameters

A kinetic study was performed using YLV and FAAF to investigate the effects of amino-acid residues adjacent to valine and phenylalanine in peptide substrates. Those pNA peptides were cleaved at valines and phenylalanines, see Table 3. With pNA, kinetic data also indicated that aliphatic and aromatic amino-acids were by far the preferred residues at positions P1 and P1/P4 for keratinases and subtilisins, respectively. All the purified SAPB enzymes exhibited the classical Michaelis-Menten kinetics for the pNA substrates used. Table 3 summarizes the *k*_cat_/*K*_m_ value of each enzyme. The findings revealed that rSAPB and SAPB-N99Y exhibited the highest *k*_cat_/*K*_m_ values of 399 min^−1^.mM^−1^ and 932.50 min^−1^.mM^−1^ when YLV was used as a specific tripeptide substrate for keratinases. YLV was also the preferred substrate for SAPB-N99Y, with *k*_cat_/*K*_m_ that were at least 100-fold higher than those observed for SAPB-L31I/T33S, SAPB-T33S, and SAPB-L31I. The differences between those enzymes were largely due to the closely similar *K*_m_ and *k*_cat_ values (Table 3), indicating that substrate binding had the greatest effect. This further confirmed the promising candidacy of SAPB-N99Y for future industrial application. Likewise, when FAAF was used as a specific tetrapeptide substrate for subtilisins, SAPB-T33S, SAPB-L31I, SAPB-L31I/T33S, and SAPB-L31I/T33S/N99Y exhibited *k*_cat_/*K*_m_ values that were 29, 38, 40, and 70 fold higher than those recorded for SAPB, respectively.

**Table 3:** Kinetic parameters of the purified wild-type and mutant SAPB enzymes with selected synthetic peptide substrates.

Substrate	SAPB enzyme	*K*_m_ (mM)	*k*_cat_ (min^−1^)	*k*_cat_/*K*_m_(min^−1^.mM^−1^)	Relative catalytic efficiency to WT
YLV	WT	0.024±0.0	9.576±0.5	399.00	1.00
	L31I	7.346±0.3	36.501±3.7	4.96	0.01
	T33S	8.344±0.4	61.364±6.5	7.35	0.01
	N99Y	0.008±0.0	7.640±0.4	932.50	2.33
	L31I/T33S	6.110±0.3	52.134±5.5	8.53	0.02
	L31I/N99Y	2.170±2.0	76.500±7.0	35.25	0.08
	T33S/N99Y	3.211±3.3	104.573±8.0	32.56	0.08
	L31I/T33S/N99Y	4.593±0.2	43.786±0.7	9.53	0.23
FAAF	WT	4.340±0.2	54.250±5.8	12.50	1.00
	L31I	0.015±0.0	7.068±3.4	471.20	37.69
	T33S	0.033±0.0	11.776±0.6	356.87	28.55
	N99Y	5.210±0.2	76.797±7.3	14.74	1.17
	L31I/T33S	0.150±0.0	75.562±7.0	503.75	40.30
	L31I/N99Y	3.001±3.1	525.535±9.9	175.12	14.01
	T33S/N99Y	2.785±2.1	390.234±9.0	140.12	11.21
	L31I/T33S/N99Y	0.003±0.0	2.629±0.8	876.66	70.13

Assays were performed using the purified proteases in 100 mM buffer containing 2 mM Ca^2+^, and 0.2 mM to 50 mM synthetic peptide substrates (YLV and FAAF) at suitable pH. The samples were incubated for 10 min at suitable temperature. Results are mean values from triplicate experiments. 1 U of protease activity was defined as the amount of enzyme that catalyses the transformation of 1 mM pNA per minute under standard assay conditions.

### 3.6. Zymogram Analysis

**Figure 1:**
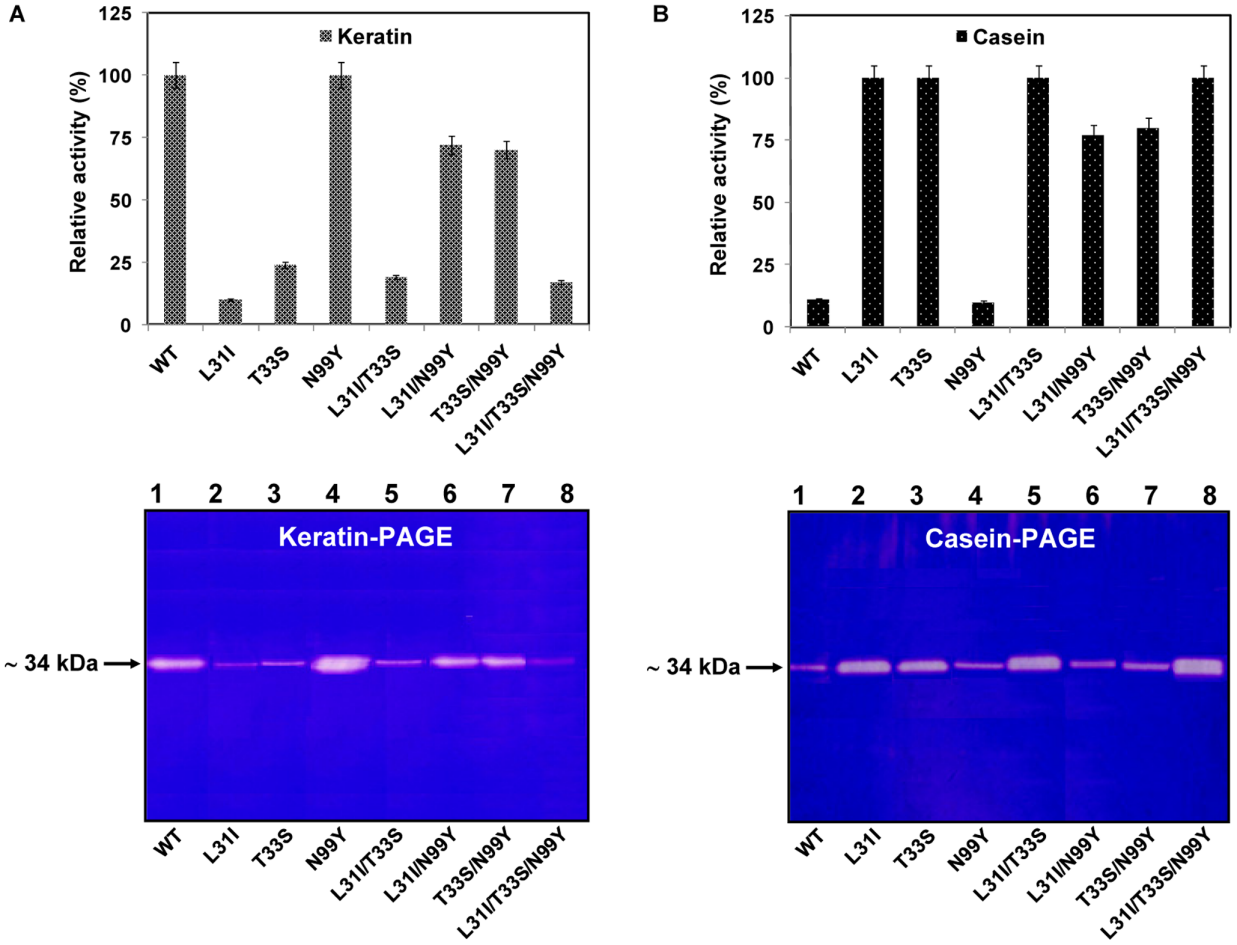
Gel-based protease activity assays (zymograms) demonstrating substrate specificity of SAPB enzymes correlated with their relative-activity. Zymogram gels were carried out under non-reducing conditions using keratin (left panel) and casein (right panel) as protein substrates and 50 µg of each purified SAPB enzyme.

In order to confirm the substrate specificity profile for SAPB enzymes, the gel-based protease activity assays (zymograms), which represent a sensitive and rapid assay method for the analysis of protease activity, were performed using gel-incorporated keratin or casein as a substrate as described in Section 2. The zymograms revealed one clear zone against the blue background for the purified samples, containing varied bands of intensity and with a molecular weight of about 34 kDa (Fig. 1). When keratin was used as substrate, the data confirmed that SAPB-N99Y exhibited the highest activity, followed by the wild-type enzyme, the double SAPB mutant L31I, and T33S. Poor to moderate keratin activities were, however, observed with SAPB-L31I, SAPB-L31I/T33S, SAPB-L31I/T33S/N99Y, and SAPB-T33S (Fig. 1A). Hence, the wild-type and SAPB-N99Y mutant enzyme displayed the profile of true keratinases. Similarly, clearer keratinolytic activities were observed in zymographic assays with the microbial keratinases KERUS [7], KERAB [22], and SAPDZ [8]. Moreover, when casein was used as a substrate, the data confirmed that SAPB-L31I/T33S/N99Y exhibited the highest activity, followed by SAPB-L31I/T33S, SAPB-L31I, and SAPB-T33S. Poor to moderate casein activities were, however, observed with wild-type and SAPB-N99Y enzymes (Fig. 1B). Accordingly, SAPB-L31I/T33S/N99Y, SAPB-L31I/T33S, SAPB-L31I, and SAPB-T33S showed the profile of true subtilisins. Likewise, clearer caseinolytic activities were visualized on zymograms with other subtilisins from *Bacillus* origins [23], [25].

Overall, the data correlated well the results obtained by the kinetics studies. The relative hydrolysis rates observed when keratin or casein were used as protein substrates and high increase of protease activity in zymograph assays elucidated the substrate specificity of the SAPB enzymes.

### 3.7. Determination of Hydrolysis Degree

**Figure 2:**
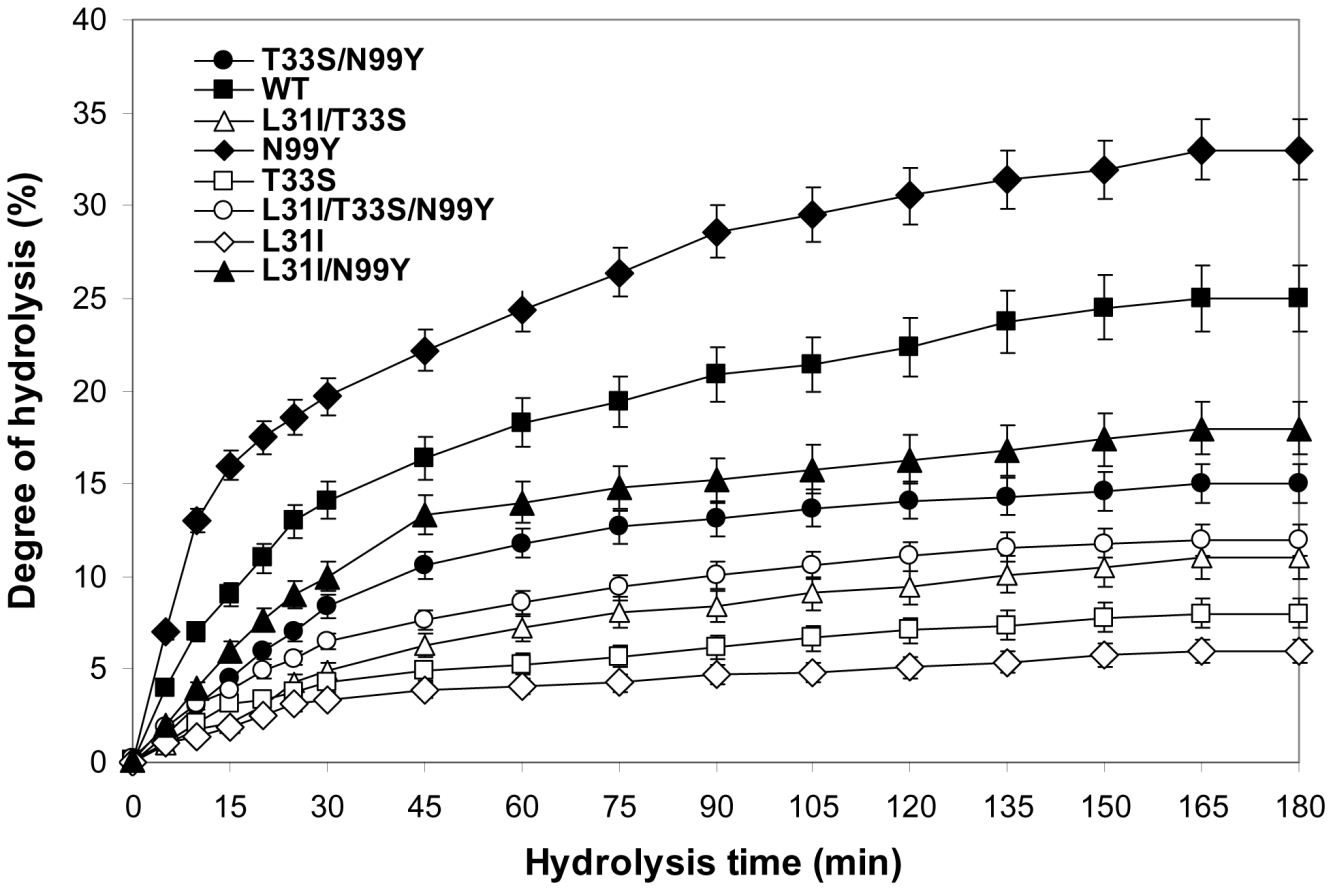
Hydrolysis curves of keratin treated with purified SAPB enzymes. The purified proteases used were: rSAPB, SAPB-L31I, SAPB-T33S, SAPB-N99Y, SAPB-L31I/T33S, SAPB-L31I/N99Y, SAPB-T33S/N99Y, and SAPB-L31I/T33S/N99Y. Each point represents the mean (n  = 3) ± standard deviation.

The hydrolysis curves of keratin azure after 3 h of incubation are shown in Fig. 2. The purified enzymes were used at the same levels of activity (2,000 U) for the production of protein hydrolysates from keratin azure and for subsequent comparisons of hydrolytic efficiencies. High hydrolysis rates were attained with keratin azure (0.4 g.l^−1^) during the first hour of incubation. The enzymatic reaction rates were noted to decrease thereafter, reaching a subsequent steady-state phase where no apparent hydrolysis took place. As shown in Fig. 2, the purified SAPB-N99Y displayed the most efficient keratinase activity (33%), followed by rSAPB (25%), with SAPB-L31I being the least efficient (6%). These findings provided further support for the usefulness of SAPB-N99Y and rSAPB in future industrial applications, particularly for upgrading the nutritional value of keratin.

### 3.8. Enzymatic Depilation of Animal Hide With SAPB Enzymes

**Figure 3:**
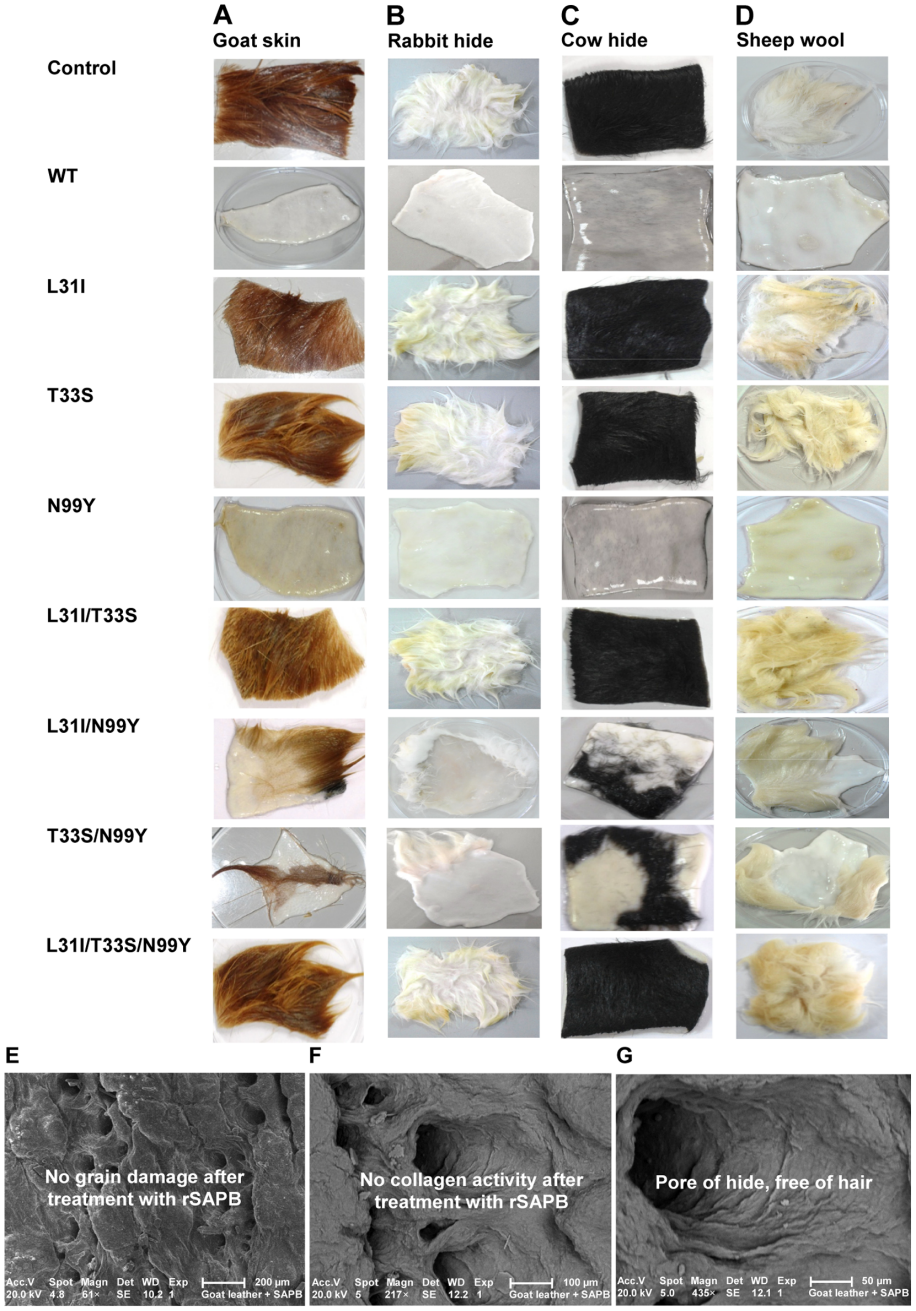
Depilation activities of SAPB enzymes on animal hides and a scanning electron micrograph-selected sectional view. SAPB enzymes were incubated for 8 h at 37°C with goat skin (A), rabbit hair (B), cow hide (C), and sheep wool (D). Every test was carried out with a control without adding enzyme. Magnification and micrographs of 61× (E), 217× (F), and 435× (G) were taken following the treatment of goat skin with rSAPB enzyme-assisted depilation. Samples show a clean pore, indicating complete removal of the hair and root.

Enzymatic depilation involves the use of keratinolytic enzymes to cleave the substances that hold the hair to the skin without causing damage to the hide. Several efforts have been directed towards developing novel proteases for animal hide depilation. Most of the proteases so far identified have a collagen degrading activity that destroys the collagen structure of the hide and are, therefore, not suitable for depilation. Accordingly, it is important to identify novel proteases that have no collagenolytic activity and, hence, efficient depilating activity [25]. In this respect, the incubation of SAPB enzymes with goat, rabbit, bovine, and sheep skin samples showed that the skins treated with rSAPB and SAPB-N99Y had their hairs removed very easily and with no visible damage on the collagen after 8 h of incubation at 37°C as compared to their control counterparts (Fig. 3). These findings provided evidence that rSAPB and SAPB-N99Y, alone, could accomplish the whole depilation process, and that SAPB-L31I/N99Y and SAPB-T33S/N99Y had moderate depilatory effects. The comparison with the specific activities illustrated in Table 1 strongly suggests a relationship between the depilation capacity and the keratin/casein ratio of each SAPB enzyme. In fact the highest ratio recorded was 4.5 for SAPB-N99Y followed by 2.5 for rSAPB, the only two enzymes showing full depilation ability. All the other mutant enzymes had moderate to low keratin/casein ratios ranging from 1.40 for SAPB-L31I/N99Y to 0.55 for SAPB-L31I. The SAPB-L31I, SAPB-T33S, SAPB-L31I/T33S/N99Y, and SAPB-L31I/T33S enzymes showed, however, keratin/casein ratios of 0.55, 0.62, 0.63, 0.70, respectively, and, hence, lacked depilation capability.

In leather processing, depilation is generally carried out at pH values between 8 and 12 [26]. The fact that SAPB-N99Y meets this operational criterion provides further support for the strong candidacy of this enzyme for future application as a hair removal agent in the leather industry. Comparatively, alkaline proteases from *B. pumilus* were reported to have high keratinolytic activity and to accomplish the depilation process on their own for bovine hair [27], cow hides [28], and goat skins [25]. Accordingly, further studies, some of which are currently underway in our laboratories, are needed to test the hide and skin depilation potential of SAPB-N99Y at an industrial scale.

### 3.9. Depilating Studies at Semi-Industrial Level With rSAPB

Preliminary experiments were set up to standardize the optimal conditions for the enzymatic depilation of goat hides by the rSAPB protease preparation using the dip method. The optimal conditions obtained were 2,000 U enzyme in water float (100% w/v) for 8 h. The process time obtained in this study was shorter when compared to previous results in the literature where the enzymatic dehairing process was fulfilled in time intervals ranging from 18 to 21 h for different animal skins [29], [30], [31], [32]. Experiments were then set up to investigate enzymatic depilation at a semi-industrial scale.

### 3.10. Visual Assessment

Visual observations revealed that the enzymatically depilated pelts from goat hides showed white surfaces having hair pores with no fine hair and that the hair was completely removed along with the epidermis. The chemically depilated pelts, on the other hand, were black and showed visible residual hairs in the hair pores. The black color of the chemically treated skins was due to the use of sulphide. The hair recovered from enzymatic depilation was intact in both skins and hides, which was attributed to the absence of the hair destructing sulphide in the depilating bath. The microscopic analysis of the hairs obtained from the enzymatic depilation of goat hides confirmed the presence of hair roots. Contrarily, the hairs from chemical depilation were pulped with damaged ends, and the hair roots were absent (data not shown). The intact hair of good quality can be a value added saleable by-product useful in organic fertilizers, poultry feed stuff, and felt manufacturing [33].

Furthermore, the chemically treated pelts were heavier and more swollen than the enzymatically treated skins, which could be ascribed to osmotic swelling brought about by the presence of lime. In the conventional process, osmotic swelling caused by exposure to high concentrations of lime leads to water absorption by pelts and, hence, the increase in weight. The hydrostatic pressure developed in the pelts by water absorption also enhances the splitting of fibre bundles. The fact that the enzyme-treated pelts were lighter can be attributed to the higher rates of interfibriller protein removal from the collagen matrix by the enzyme. Due to the elimination of lime from the process, the osmotic swelling of the enzyme-treated skins was not adequate and was, however, adjusted in subsequent steps of leather processing.

### 3.11. SEM Analysis

The enzyme-treated samples from the “SO. SA. CUIR” leather tannery (M’Saken, Sousse, Tunisia) were subjected to morphological studies using SEM; the photomicrographs are shown in Figs. 2E–G. Compared to the conventional systems, the complete absence of the above structural features was observed in the depilated pelts of goat hides obtained after enzymatic dehairing. While the sections from the chemically depilated pelt showed remnants of hair root in the hair follicle, those from the enzymatically treated ones showed that the hair was completely removed from the root. The collagen structure in the enzyme-treated pelts was intact with no apparent damage to the collagen fibres. The inactivity of protease on skin collagen is one of the prerequisite for its application in depilation. Some of the proteases previously reported for depilation need controlled application for they were shown to bring relative degrees of damage to the collagen at the grain layer, which can impart unfavorable properties to finished leather [34], [35].

Information on grain surface of depilated pelt and dyed crust was obtained by SEM analysis (Figs. 2E–G). The findings revealed that the grain structure of the enzymatically depilated group was cleaner and that their surfaces were smoother, with opened collagen fibre bundles, than the chemically dehaired group. Moreover, the hair pores on the enzyme-treated pelts did not show residual hair, indicating hair removal from the root. The opening of the collagen fibre structure was complete and regular in the enzymatically dehaired pelts, with the degradation of the interfibrillar substances. The extent of interfibrillar substance removal is directly proportional to the degree of collagen fibre bundle opening [36]. This could further accelerate the penetration of protease through the collagen matrix to act upon anchoring proteins around the hair follicle and eventually facilitate hair removal. The enzyme-treated samples also showed a clear surface with no deposition of foreign particles or grain damage due to degradation of the adhering non-structural protein materials surrounding the hair roots, thus resulting in complete hair removal.

### 3.12. Physical and Chemical Properties of Dyed Crust

The physical testing of the dyed crust from the rSAPB-treated goat hides showed that enzymatic treatment did not affect the strength properties of the leather adversely. The physical characteristics of the enzyme-treated dyed crusts *viz.* tear strength, elongation percentage, tensile strength, and stitch tear resistance were in good agreement with those of the conventional process (Table 4). This was due to higher fibre opening in the enzyme treated pelts, which resulted in higher degrees of softness for the enzyme-treated leather. The softness of leathers is related to the opening up of fibre bundles. The crusts of well-opened fibre structure show more degrees of softness than the moderately or incompletely opened up ones [37].

**Table 4:** Physico-chemical characteristics of dyed crusts produced from goat skins.

Property		Method	
		Conventional	Enzymatic
Physical	Tensile strength (N.mm^−2^)	19.00±0.6	27.62±1.0
	Elongation at break (%)	17.00±0.5	38.67±1.2
	Tear strength (N.mm^−1^)	35.50±1.1	54.75±2.1
	Stitch tear resistance (N.mm^−1^)	51.37±2.0	20.31±0.7
Chemical	Hide substance (%)	20.10±0.7	13.60±0.4
	Oils and fats (%)	1.95±0.0	1.70±0.0
	Cr_2_O_3_ (%)	7.15±0.2	5.32±0.2
	pH	3.63±0.1	3.26±0.1

Values represent means of three samples from three sets of experiments, and ± standard errors are reported.

The analysis of the chemical properties of the dyed crusts obtained from the enzymatic and chemical treatments showed similar patterns (Table 4). The amount of hide substance recorded in the enzyme-treated dyed crust from goat hides was 13.6%, which was approximately equal to the amount registered for the chemically treated group (20.1%), indicating that the collagen content was not affected by enzyme treatment. The chrome content in enzyme-treated goat leather (5.32% for buffalo and goat skins, respectively) was higher than that of the chemical control (7.15%), which indicated good fibre opening. The finished leathers were also assessed for dyeing properties, softness and general appearance. The results revealed that the enzyme-treated leather was comparable to the chemically treated one. The oil and fat content of the enzyme-treated leather was also comparable to that of the chemically treated leather. In fact, grease gives suppleness and handle to leather. While too little grease results in hard leather that will tend to crack, too much grease not only makes the leather feel greasy but creates subsequent problems during the manufacturing process.

### 3.13. Analysis of Environmental Parameters

Pretanning processes generally account for 70–80% of the total pollution load from the whole leather making process [38], and the reduction of pollution at this stage can be considered critical for making the whole process ecofriendly. Accordingly, the enzymatic depilation process of skins and hides was assessed in terms of pollution control parameters, including BOD, COD, TSS, sulphide, and calcium (Table 5). The effluent collected after the depilation step from the enzyme-treated group contained dislodged intact hair and epidermal substances whereas the effluent from chemical depilation consisted of lime, sulphide, epidermal substances, and degraded pulped hair. When compared to chemically treated controls, the total Kjeldahl nitrogen (TKN) and TSS in effluents from the enzymatic depilation of goat hides were reduced by 59–77%. This can be attributed to the elimination of lime from the process which resulted into sludge formation. The BOD_5_ and COD of effluents from the enzymatic depilation of goat hides were reduced by approximately 38%. This significant reduction in BOD and COD was due to the removal of lime and hair degrading sulphide from the process. Degraded pulped hair, rich in nitrogen, also contributes to high BOD_5_ and COD in the effluent from conventional processes. The elimination of sulphide in enzymatic depilation facilitated the recovery of intact hair, thus leading to low COD values in the effluent. In similar studies, 25% reduction in BOD and COD was reported by Sundarajan et al. [39] and approximately 50% reduction by Dayanandan et al. [30]. Moreover, as lime was eliminated from the enzymatic process, it did not generate an alkaline effluent, and the pH of the effluent was almost neutral, indicating its lower levels of toxicity.

**Table 5:** Analysis of pollution parameters from the effluent of depilating processes of goat skins.

Parameter		Conventional	Enzymatic	Reduction (%)
BOD_5_ (mg.l^−1^)	Bath depilation	30246	19000	38
	Washing bath after depilation	9986	4174	59
COD (mg.l^−1^)	Bath depilation	44160	27740	48
	Washing bath after depilation	14580	6095	59
TSS (mg.ml^−1^)		58300	13900	77
TKN (mg.l^−1^)		10000	4100	59
Sulphide (mg.ml^−1^)		2680	–	100
Calcium (mg.ml^−1^)		4200	–	100
Conductivity (ms.cm^−1^)		28	17	40
pH		13	6.5	50

Results were average of three different sets of experiments.

### 3.14. Structural Interpretation

**Figure 4:**
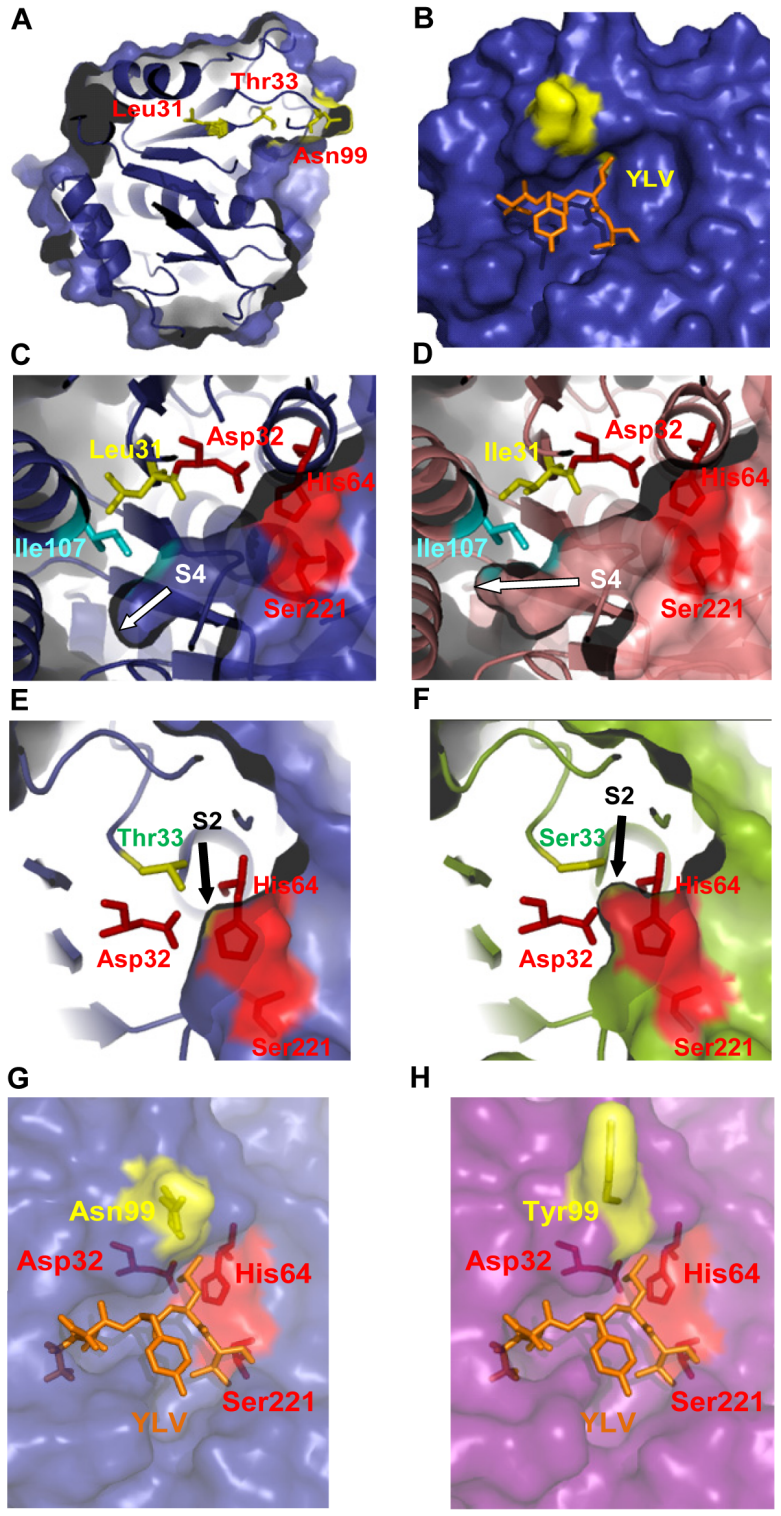
Structural interpretation. (A) SAPB model showing the positions of the mutated amino-acids. (B) Surface representation of SAPB which the YLV tripeptide synthetic substrate, shown in orange sticks, has docked. Close up views of (C) the catalytic cavity and Leu31, showing a superposition of surface and ribbon representations of the SAPB model, (D) the catalytic cavity and the Ile31 residue in the SAPB-L31I model. (E) Thr33 in the SAPB model showing a superposition of surface and ribbon representations, and (F) Ser33 in the SAPB-T33S model showing a superposition of surface and ribbon representations. (G) Asn99 in the SAPB model showing a superposition of surface and ribbon representations, and (H) Tyr99 in the SAPB-N99Y model showing a superposition of surface and ribbon representations. The mutated residues are shown in yellow sticks and surfaces. These figures were prepared using the PyMol software (http://www.pymol.org).

In the absence of an experimental three-dimensional structure, the generation of a model structure through use of known homologous enzyme structures is helpful for understanding the roles of various mutations in improving the characteristics of enzymes. Though no crystal structure is currently available for SAPB, the 3D structure of this enzyme is likely to be similar to that of the bacilli subtilisins E, BPN, and Carlsberg, which was evidenced by their significant sequence identities of 69%, 66%, and 65%, respectively. The 3D structure of the complex subtilisin E-pro-peptide [40] was used as a template to build a model structure for the SAPB-WT [11] and SAPB mutant enzymes (Fig. 4). The possible effects of the three single mutations at positions 31, 33, and 99 in the SAPB enzyme were studied, using the homology-model structure along with the known structure of subtilisin E (PDB-code 1SCJ) from *B. subtilis* [40]. In fact, the importance of residues Leu31 and Thr33 with regard to specific activity and enzymes’ pH and temperature profile has already been reported in the literature [11], [24]. However, and to the authors’ knowledge, no previous work has reported on the potential roles of those two residues bordering the catalytic residue Asp32 in substrate recognition and animal hide enzymatic depilation.

The generated SAPB models showed that Leu31 was located at the C-terminal end of the β1 sheet, which was close to the catalytic cavity (Figs. 3ABC). According to the model, Leu31 was not directly involved in substrate binding but involved in a network of hydrophobic interactions that could have shaped the active site of the enzyme, especially with Ile107 (Figs. 3AD). This latter residue was directly implicated in the binding cleft subsite S4. According to the model of SAPB-L31I, an isoleucine in position 31 would not have established this hydrophobic contact. As a result, the catalytic cavity might have been enlarged (Fig. 4D) fitting more easily a bulky residue, such as phenylalanine, which is present in most subtilases [41]. The kinetic parameters of this mutant corroborated this hypothesis since the mutant enzyme became more specific for a substrate that displayed a phenylalanine (FAAF) in position P4.

Concerning the T33S mutation, Thr33 was directly involved in the substrate binding site being located at the surface of subsite S2 (Figs. 3AE). In the SAPB model, residue P2 would fit exactly the cavity site and establish a hydrogen bond with the catalytic residue Asp32. The mutant enzyme model shown in Figs. 4AF, suggests that subsite S2 was also enlarged and became more selective. Though the S2 binding site would be expected to be better adapted for large side chains, the opposite effect was observed. In fact, substrate docking simulations with the best substrate of the SAPB-T33S model (FAAF) revealed that it bound in a way that was similar to that of the YLV tripeptide synthetic substrate in the SAPB model (Fig. 4B), with the backbone trajectories of the two peptides being almost identical. More interestingly, the S2 binding pocket was noted to remain largely unoccupied by the methyl side chain of the P2 alanine residue, and, consequently, no hydrogen bonds would be established between the catalytic Asp32 and the residue in P2. In fact, most of studies so far performed on the role of catalytic subsites (S1, S2, S3, S4) concluded that the S2 site may not be vital for defining substrate specificity as compared to the S1 and S4 sites [23]. The findings of the present study, however, clearly demonstrated the important role of subsite S2 in substrate specificity, which was further consolidated by the fact that the catalytic residues His64 and Asp32 were both involved in maintaining the residue in position P2. Additionally, since Leu31 and Thr33 were close to the catalytic Asp32, this change could be attributed to the enhanced keratin hydrolysis, which in turn could explain the unchanged kinetic parameters of the SAPB-L31I and SAPB-T33S mutants when compared to the wild-type enzyme. The preference for longer substrates at both sides of the scissile peptide bond suggests the suitability of keratinases for the conversion of native and complex substrates. In fact, the cleavage of peptide bonds in the compact keratin molecules is difficult due to the restricted enzyme-substrate. Therefore, the hydrolysing ability of keratinolytic proteases may be due to their ability and specificity to bind to compact substrates, and more open active sites.

Concerning Asn99, which was located in a turn preceding β-strand 4 in SAPB (Fig. 4GH), the replacement Asn/Tyr in SAPB-N99Y influenced the shape and flexibility of the gate wall at the substrate binding cleft, which resulted in high substrate selectivity [11], [24]. Thus, the polarity of the amino-acid at position 99 was an important factor for substrate affinity due to its localization next to the substrate binding cleft along with its ability to perform π-π interactions with the nitroanilide parts of the synthetic pNA substrates.

### Conclusions

In this study, extracellular alkaline rSAPB protease from *B. subtilis* DB430/pNZ1 was purified and characterized, and an ecofriendly enzymatic depilation process was developed for goat hides, completely eliminating the use of lime and sulphide. The process resulted in the complete removal of hair with well opened collagen fibre bundles, which was further confirmed by SEM analysis. The dyed crusts produced by enzymatic depilation displayed better physico-chemical properties when compared to the chemical group. Moreover the process resulted in a significant reduction of pollution parameters, making it ecofriendly. The results from the skin application trials at industrial level supported the promising biotechnological potential of this enzyme for the ecofriendly depilation of animal skins in the leather industry without affecting the quality of the leathers produced. This study also demonstrated that the Leu31 and Thr33 residues play important roles in substrate recognition and hydrolysis. The findings from molecular modeling analyses indicated that L31I and T33S mutations could directly impinge on the dimensions of S2 and S4 substrate binding sites affecting enzyme specificity. They clearly demonstrated that the engineering of kinetic performances of enzymes of interest is not restricted to the amino-acids of the catalytic cluster but relates to the hydrophobic environments near the active site. This study is the first to demonstrate the promising keratinase and depilation activities of SAPB and the transformation of subtilisin into keratinase.
